# A Telegram-based behavioral intervention for smoking cessation after myocardial infarction: A study protocol of a pragmatic, single-blind, randomized trial

**DOI:** 10.18332/tpc/218842

**Published:** 2026-06-30

**Authors:** Mikhail Kuznetsov, Artemiy Okhotin, Dmitry Sergeev

**Affiliations:** 1 ITMO UniversitySt. PetersburgRussia; 2 Ulyanovsk Regional Clinical HospitalUlyanovskRussia; 3 Medical Faculty Heidelberg, University of HeidelbergHeidelbergGermany; 4 German Cancer Research Center (DKFZ)HeidelbergGermany

**Keywords:** myocardial infarction, smoking cessation, randomized controlled trial, digital health, secondary prevention

## Abstract

Smoking cessation after myocardial infarction (MI) is critical, yet many patients relapse soon after discharge, especially where structured follow-up is limited. Telegram provides a low-cost tool for implementing chatbot-based behavioral support that hospitals can run with minimal technical resources. The aim is to conduct TELEGRAM-MI trial tests to ascertain whether a 6 month Telegram-based chatbot improves cessation outcomes compared with routine discharge advice provided by cardiology staff. This pragmatic, single-blind, randomized trial, starting in 2026 at a regional clinical hospital in Russia, will enroll adult current cigarette smokers hospitalized with acute MI who use Telegram. Participants are randomized 1 : 1 to routine discharge advice plus a passive bot (control) or to the same advice plus an active 6 month behavioral support bot. The intervention includes motivational messages, craving-management tools, an SOS function, weekly check-ins, and relapse-prevention sequences. The primary outcome is 30 day point prevalence abstinence at 6 months, verified by a blinded assessor and a relative. Secondary outcomes include 7 day abstinence at 1 and 3 months, changes in cigarettes/day, nicotine dependence, motivation, engagement, and time to relapse. Analysis will follow intention-to-treat principles. Recruitment begins in January 2026. Results are expected after completion of the 6 month follow-up in October 2026. The intervention could enhance cessation, sustain recovery after MI, and offer a practical way to extend secondary prevention beyond the hospital site.

## Introduction

Tobacco smoking remains a major global health problem, and helping people quit is difficult even in settings with established cessation programs^1,2^. For patients who survive a myocardial infarction (MI), the cardiac event often becomes a strong trigger to stop smoking, but maintaining abstinence after discharge remains challenging^3^. In Russia, usual care typically consists of brief physician advice at discharge, with almost no structured long-term follow-up. As a result, many patients relapse shortly after returning home, despite the high risk of recurrent cardiac events^4^. At the same time, messaging platforms such as Telegram are used daily by millions of people and can deliver ongoing behavioral support at very low cost^5^. Most hospitals do not have established digital follow-up systems. Still, automated chatbots offer a practical way to extend clinical care beyond the hospital site, and help patients stay motivated during recovery^6^. Because Telegram allows rapid deployment of automated support tools without collecting sensitive personal data, it represents a promising channel for scalable digital health interventions^7^. Based on this opportunity, we designed the TELEGRAM-MI trial to test whether a ^,^6-month^6,8^ Telegram-based support program can improve smoking cessation outcomes in MI survivors compared with usual care only.

## Methods

### Study design

The TELEGRAM-MI trial is a pragmatic, randomized controlled trial with blinded outcome assessment, initially conducted as a single-center study with a protocol structure that allows for potential multicenter expansion. Participants will be randomized in a 1 : 1 ratio to the intervention or control group. The trial will be conducted in inpatient cardiology departments at the Ulyanovsk Regional Clinical Hospital. The pragmatic design aims to assess the intervention’s effectiveness in real-world clinical practice. The protocol was approved by the Local Ethics Committee of the clinical center (Ulyanovsk regional hospital, #PN0104122025, 04.12.2025) and registered on ClinicalTrials.gov (NCT07230249). Written informed consent will be obtained from all participants.

### Eligibility criteria

Participants will be identified, screened, and enrolled during hospitalization for acute MI.

### Inclusion criteria

The inclusion criteria will be: 1) adults aged ≥18 years; 2) a confirmed diagnosis of acute MI and being current cigarette smokers (≥1 cigarette/day in the month before hospitalization – only cigarette smoking will be considered to ensure a homogeneous study population); 3) regular user of Telegram on a personal smartphone; 4) fluent in Russian; and 5) willing to provide written informed consent and contact details of a close relative/cohabitant for outcome verification.

### Exclusion criteria

Exclusion criteria will be: 1) potential smoking cessation >1 month before MI; 2) severe physical or cognitive impairment preventing smartphone use; 3) severe, uncontrolled mental illness impeding protocol adherence; 4) severe comorbid condition with a poor short-term prognosis; and e) unwillingness to provide a relative’s contact for verification.

### Recruitment, consent, and randomization

**Figure 1:**
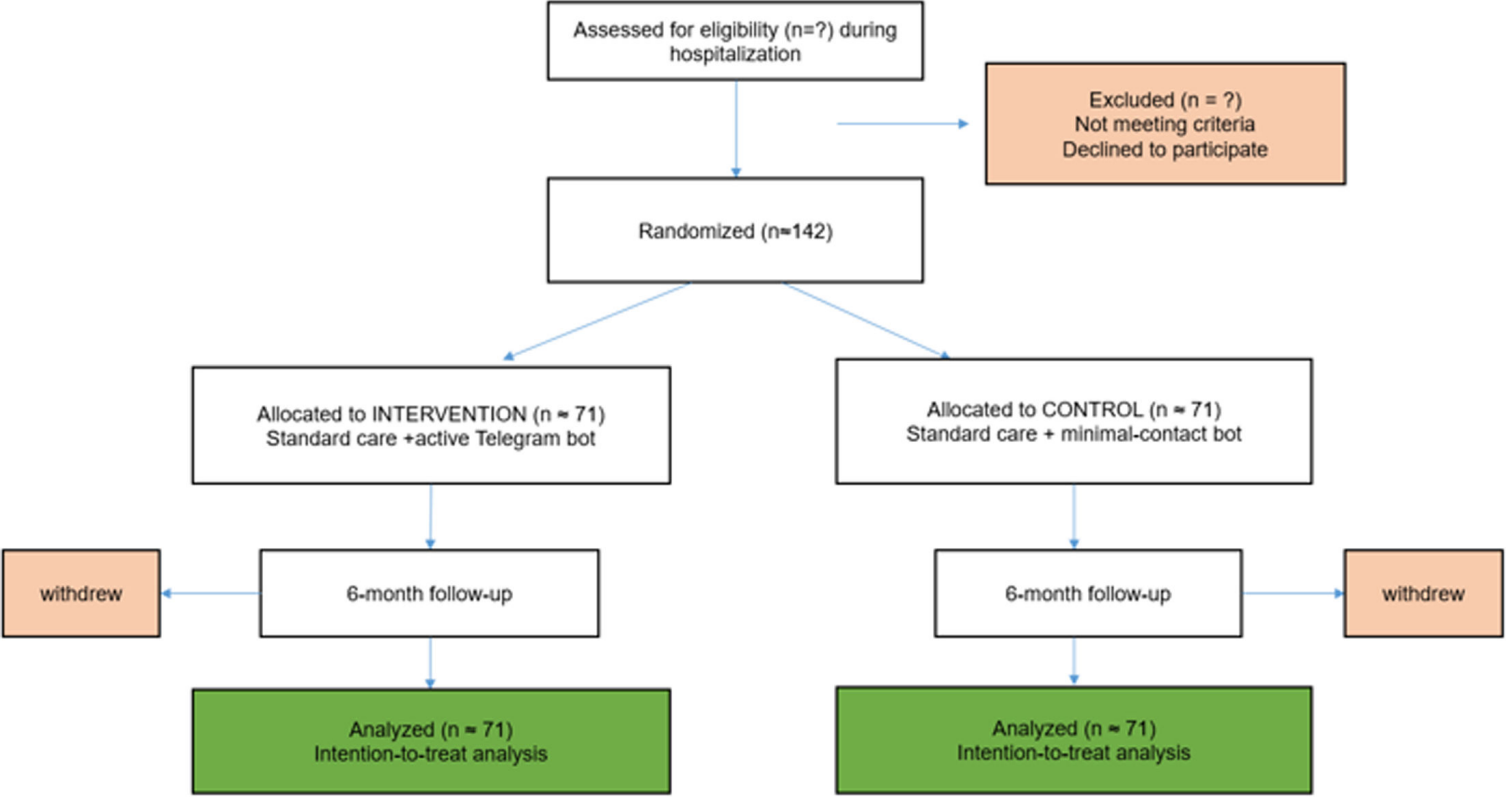
Participant flow diagram of the TELEGRAM-MI pragmatic, single-blind, randomized trail conducted in a regional clinical hospital in Russia (2026)

A treating cardiologist or study nurse will screen potential participants. After providing written informed consent, participants will be assisted in connecting to the study’s Telegram bot before discharge. Randomization will occur automatically within the bot upon completion of the baseline assessment. The allocation sequence (1 : 1) will be computer-generated with variable block sizes by an independent statistician using R software and integrated into the bot’s backend. Allocation concealment is ensured, as the enrolling clinician will have no access to the sequence; the group assignment is revealed only by the bot after the baseline survey is finalized. The flow of participants through the study is illustrated in Figure 1.

### Blinding

While participants cannot be fully blinded to the intervention, they are not explicitly informed of their group assignment. As well, outcome assessment is blinded (single-blind). The assessor conducting the 6 month telephone verification will be unaware of group allocation and will have no access to bot interaction data.

### Interventions

#### Control group (usual care + passive bot)

Participants receive usual care (advice to quit and pharmacotherapy at the discretion of the physician) and are connected to a Telegram bot used only for completing baseline and follow-up assessments (1, 3, and 6 months). The control bot sends no smoking-cessation advice, motivational content, or personalized feedback.

#### Intervention group (usual care + active telegram support)

Participants received a 6 month behavioral intervention via a Telegram chatbot. The bot delivered content across several categories (e.g. motivational^9^, behavioral coping strategies^10^, check-in prompts) following a tapered frequency schedule: daily messages during months 1–2, reducing to three times per week in months 3–4, and weekly in months 5–6. Personalization was rule-based: initial intensity was informed by the baseline Fagerström score, and subsequent support was dynamically adapted based on weekly check-in responses (e.g. increased outreach following reports of high cravings or lapse). Bot script will be shared.

### Outcomes and assessments

#### Primary outcome

The primary outcome will be 30 day point prevalence abstinence (PPA) at 6 months, defined as self-reported non-smoking via bot for the preceding 30 days corroborated by a relative/cohabitant report during a blinded telephone interview. Participants with missing data or data not corroborated by a relative after two attempts will be considered smoking.

#### Secondary outcomes

Secondary outcomes will include: 7 day point prevalence abstinence (via bot) at 1 and 3 months; change in cigarettes per day; change in Fagerström test for nicotine dependence (FTND) score; change in Prochaska’s Motivation to Quit Smoking Questionnaire score (0–8); engagement metrics (weekly check-in completion rate, SOS-button presses); intervention acceptability/satisfaction score (12-item Likert scale); and time to first relapse (any cigarette) after ≥24 h of abstinence. Data collection, time points, mode of assessment, and the questionnaires administered are summarized in Table 1. Missing data will be reported and accounted for in the analysis (for secondary outcomes based on available cases).

**Table 1:** Schedule of outcomes and assessments in the TELEGRAM-MI pragmatic randomized trial testing a Telegram-based intervention for smoking cessation after myocardial infarction, Russia, 2026

Outcome	Type	Measurement tool/ source	Baseline	1 **month**	3 **months**	6 **months**
30 day point prevalence abstinence	Primary	Blinded phone interview + relative confirmation				X
7 day point prevalence abstinence	Secondary	Telegram bot self-report		X	X	X
Cigarettes per day	Secondary	Telegram bot self-report	X	X	X	X
FTND score	Secondary	Fagerström test for nicotine dependence (bot)	X			X
Motivation to quit^a^	Secondary	Self-rated scale via Telegram bot	X			X
Engagement metrics^b^	Secondary	Automatically logged by Telegram bot		X	X	X
Acceptability/ satisfaction score	Secondary	Custom questionnaire via Telegram bot (intervention only)				X
Time to first relapse	Secondary	Continuous monitoring via weekly check-ins (bot)		X	X	X

a Prochaska questionnaire (0–6).

b Check-ins, openings etc.

FTND: Fagerström test for nicotine dependence.

### Sample size calculation

Assuming 6 month abstinence rates of 10% (control)^4^ and 30% (intervention), 120 participants (60/group) are needed for 80% power with a two-sided alpha of 0.05. Allowing for a 15% dropout rate, the target sample size is 142 (71/group). The assumptions are based on prior evidence, where abstinence after brief advice is around 10%^9^, and a larger effect is considered plausible due to the acute motivational impact of myocardial infarction^11^.

### Statistical analysis

Analyses will follow the intention-to-treat principle, with participants lost to follow-up considered smokers for the primary outcome. Baseline characteristics will be presented descriptively in Table 2^6^. The primary analysis will compare 6 month abstinence rates using a chi-squared test, reporting absolute risk difference and relative risk (RR) with 95% confidence intervals (CIs). Continuous outcomes will be analyzed using linear mixed models. Time to first relapse will be analyzed with Kaplan-Meier curves and Cox regression. Exploratory analyses will use logistic regression to assess associations between baseline factors, engagement, and cessation. A per-protocol secondary analysis will include intervention-group participants who completed ≥60% of weekly check-ins during the first 3 months and had their final assessment within 6 months±3 weeks; this criterion does not apply to the control group.

**Table 2:** Baseline demographic and clinical characteristics of TELEGRAM-MI participants by trial arm (template for future Table)

Characteristics	Total	Intervention	Control
**Sociodemographic**			
Age (years), mean (SD)			
Age≥65 years, n (%)			
Male, n (%)			
**Smoking-related**			
Cigarettes per day, mean (SD)			
Years of smoking, mean (SD)			
FTND score (0–10), mean (SD)			
FTND≥6 (high dependence), n (%)			
Previous quit attempts (past year), n (%)			
Motivation to quit^a^, mean (SD)			
**Technology use**			
Daily Telegram use, n (%)			

a Prochaska questionnaire (0–6)

FTND: Fagerström test for nicotine dependence.

### Data management

Data will be stored on a secure, encrypted server. Because the Telegram bot interacts with participants via usernames rather than accessing personal phone numbers, the primary dataset is inherently pseudonymized. Identifiable data will be kept separate in a password-protected file with restricted access. No formal Data Monitoring Committee is planned due to low risk. Interim analyses are not planned.

## Discussion

Although several tobacco cessation apps and chatbots already exist, none is designed for the specific needs of MI survivors, a group for whom smoking cessation is often driven not by personal readiness but by the acute medical crisis and forced withdrawal during hospitalization^9^. This population requires sustained, structured support after discharge, yet most existing digital tools operate independently of healthcare systems and are not integrated into routine cardiac follow-up^9^. A key innovation of the TELEGRAM-MI trial is that it tests a model in which hospitals themselves can remotely support patients using a low-cost digital tool built on a widely used social messaging platform. Unlike commercial apps, a Telegram-based chatbot can be developed, deployed, and maintained directly by clinical teams without collecting sensitive personal data, making it feasible for public hospitals with limited technical infrastructure. To our knowledge, no trial has evaluated a messenger-based automated support program specifically for post-MI patients or explored whether a hospital-operated chatbot can extend secondary prevention beyond the hospital walls. This approach represents a novel direction for digital cardiac rehabilitation and offers a scalable strategy for long-term tobacco cessation support in high-risk patients.

### Strengths and limitations

This trial has several key strengths. First, its pragmatic design embeds the intervention within routine cardiology care, enhancing the real-world applicability and scalability of findings. Second, the use of a widely adopted messaging platform in this country (Telegram) minimizes barriers to participant training and leverages a familiar technology to sustain engagement. Third, the methodology incorporates blinded outcome assessment with external verification (relative report), reducing detection bias for the primary endpoint.

Several limitations must be acknowledged. The primary reliance on self-reported abstinence, despite external verification, is subject to potential social desirability bias in the absence of biochemical validation (e.g. cotinine testing). This methodological choice was guided by the pragmatic, remote nature of the trial to maximize follow-up rates and reduce participant burden. Second, the eligibility criterion requiring Telegram use excludes smokers who do not use this platform or smartphones, which may limit the generalizability of the results to the entire post-MI smoking population and could introduce selection bias, potentially excluding people with lower levels of education and income. Third, the initial single-center recruitment may limit the geographical and clinical diversity of the sample, although the protocol allows for multicenter expansion. Additionally, exclusion of other tobacco products limits the generalizability of the findings.

## Conclusions

If effective, this intervention offers a scalable, low-cost model for integrating behavioral support into cardiac rehabilitation in resource-constrained settings. It may also provide sustained cessation support beyond the brief quit advice given in hospital and help shape national cessation strategies by showing whether a simple digital tool can improve quit rates in a high-risk group. Future research should focus on implementation studies, trials with biochemical validation, adaptation to other risk factors, and use in other chronic conditions such as COPD or diabetes. Protocol version 1.0 is dated 27 October 2025. Recruitment is planned to commence in January 2026, with primary completion (final follow-up at 6 months) estimated for October 2026.
